# Distribution and predictors of F-18-FDG uptake values of non-malignant cervical lymph nodes in pediatric patients

**DOI:** 10.1186/s13550-024-01110-9

**Published:** 2024-05-29

**Authors:** Jeremy Godefroy, Raphael Godefroy, Koral Vedder, Yair Altura, Alexandre Chicheportiche, Simona Ben-Haim, Gal Goldstein

**Affiliations:** 1 Department of Medical Biophysics and Nuclear Medicine, Hadassah Medical Center, Jerusalem, Israel; 2 Department of Economics, Universite de Montreal, Montreal, QC Canada; 3 Faculty of Medicine, Hebrew University of Jerusalem, Jerusalem, Israel; 4 University College London, London, UK; 5 The Dyna and Fala Weinstock Department of Pediatric Hematology-Oncology, Hadassah Hebrew University Medical Center, Jerusalem, Israel

**Keywords:** Positron emission tomography, Cervical lymph nodes, Children, Tonsil, Deauville score

## Abstract

**Background:**

F-18-flurodeoxyglucose (FDG) PET/CT is routinely used for staging, evaluation of response to treatment and follow-up of most pediatric malignancies. Cervical lymph nodes can be involved in some pediatric malignancies, but increased uptake in non-malignant cervical lymph nodes is not exceptional in this population. The aim of the present study is to identify predictors of the maximum uptake in non-malignant cervical lymph nodes in the pediatric population.

**Methods:**

191 FDG PET/CT studies of pediatric patients without malignant involvement of cervical lymph nodes were retrospectively reviewed. The maximal Standard Uptake Value in the hottest cervical lymph node (SUVmax_CLN_), as well as demographic, technical and imaging variables were recorded. The predictive effect of those variables on SUVmax_CLN_ was estimated using linear regression models.

**Results:**

Increased FDG activity in cervical nodes was observed in 136/191 studies (71%). The mean SUVmax_CLN_ was 2.2 ± 1.3. Ipsilateral palatine tonsil SUVmax, mean liver uptake, and treatment status were all statistically significant predictors of SUVmax_CLN_. However, in multivariate regression analysis, only ipsilateral palatine tonsil SUVmax was found to be significant. In addition, SUVmax_CLN_ was greater than the mean liver uptake in 50% of all studies. This proportion was higher in younger children, reaching 77% of studies of children younger than six years.

**Conclusion:**

SUVmax in ipsilateral palatine tonsil is a strong predictor of the maximal uptake value of non-malignant cervical lymph nodes in children. The intensity of uptake in non-malignant cervical lymph nodes is frequently higher than liver uptake in children, and this tendency increases for younger patients.

**Trial was registered:**

In the internal hospital registry under TRN 0209-22-HMO on date 23.04.2022.

## Background

F-18-flurodeoxyglucose (FDG) PET/CT is routinely used for staging, evaluation of response to treatment and follow-up of most pediatric malignancies [1]. Lymphoma represents about 20% of all pediatric malignancies and frequently involves cervical lymph nodes, especially Hodgkin lymphoma [2]. Among solid tumors, rhabdomyosarcomas represent about 4% of all pediatric malignancies, and about half of them are located in the head and neck area, with a 10–20% risk of regional lymph node disease [3]. Accurate staging of the cervical lymphatic stations is necessary to adequately tailor therapeutic strategies, and to determine whether response to treatment is satisfactory. For Hodgkin lymphoma for instance, negative PET/CT after two cycles of chemotherapy has been shown to be predictive of a good prognosis and to alleviate in some cases the need for radiotherapy [4]. However, FDG increased uptake is not specific for malignancy and is common in reactive lymph nodes in the context of seasonal viral pharyngeal infections, particularly common in the pediatric populations [5]. After treatment, increased FDG uptake may be encountered in other benign conditions such as lymph node hyperplasia [6]. CT-measured size of the cervical lymph nodes is also highly nonspecific due to the variability of this measure [7]. Criteria based on the SUV thus lack specificity: a “hot” cervical lymph node is a common finding without clear cut-off uptake values for positivity. The objective of the present study is to analyze the distribution of the SUVmax of non-malignant cervical lymph nodes in a pediatric population and to identify predictor variables of that value. The ultimate aim is to estimate the expected uptake value of non-malignant cervical lymph nodes, improving the accuracy of the interpretation of a “hot” cervical lymph node. The choice of the potential predictor variables is guided by clinical considerations. It should be easy to obtain, not observer-dependent, and not influenced by potential malignant involvement of the cervical lymph nodes. In addition to basic demographic predictors, we assumed that for most clinical settings, the uptake in the liver and in the palatine tonsils are imaging parameters that fill those requirements and therefore studied their influence on the value of uptake on non-malignant cervical lymph nodes.

## Materials and methods

### Patients

Inclusion and exclusion criteria are listed in Table 1.

**Table 1:** Inclusion and exclusion criteria

Inclusion criteria	Exclusion criteria
• Under 20 years of age (s)	• For solid tumors, primary tumor in the head and neck area (p)
• Referred by the pediatric haemato-oncology department of our institution (p)	• For lymphoma patients, staging studies (s)
	• Suspected involvement of cervical lymph nodes per PET/CT (s)
	• No imaging or clinical follow-up 6 months after included PET/CT (s)
	• Suspected relapse in the neck area during 6 months follow-up (s)
	• Tracheostomy (s)
	• Referred for non-oncological reason (fever of unknown origin, infectious diseases) (s)
	• Prominent brown fat uptake in the neck (s)
	• Motion artifacts in the neck area (s)
	• Misregistration PET and CT (s)

Criteria are either per patients(p) or per PET/CT study (s)

All FDG PET/CT studies performed from January 2015 to January 2022 of patients up to 20 years of age referred by the pediatric haemato-oncology department of our institution were eligible for inclusion. To include only the patients with benign cervical lymph nodes several steps of selection were used. Only patients with available clinical or imaging follow-up data for a minimum of 6 months after the PET/CT were included. For patients with solid tumors (154 out of 191 studies), patients with primary tumor in the head and neck area (e.g. head and neck rhabdomyosarcoma) were excluded. For none of the remaining patients there was imaging evidence of suspicion of cervical lymph nodes involvement as reported by interpreting physicians (in the vast majority of the cases not one of the two physicians involved in the data acquisition). Patients with evidence of or even suspected relapse in the neck in the six months after the PET/CT were excluded. However, for none of the included studies pathological data were available for the cervical lymph nodes (since biopsy is done only for suspicious cases, that were excluded from the study).

For lymphoma patients (37 studies out of 191), for whom there is a theoretical risk of infra-imaging disease involvement that may disappear under treatment, we excluded all studies performed at presentation. Studies with suspicion of cervical lymph nodes involvement either as reported by the interpreting physician or by one of the two physicians involved in the data acquisition of the study were excluded. In a similar fashion to the selection steps used for solid tumor patients, we excluded studies without a 6 month follow up period showing no clinical or imaging evidence of disease in the neck.

Patients with benign conditions, possibly interfering with uptake in cervical lymph nodes, such as tracheostomy and fever of unknown origin, as well as patients with prominent brown fat uptake in the neck were also excluded. PET/CT studies with motion or misregistration of the head or neck were excluded.

Patients were considered under active treatment if they received their last course of chemotherapy less than 30 days before the PET/CT study.

The study has been approved by the institutional review board, and the need for written informed consent was waived.

### Imaging

FDG PET/CT was performed according to the EANM guidelines [1, 8]. When needed sedation was done during the scan. Four dedicated PET/CT systems were used along the study: Discovery ST (GE Medical Systems, Milwaukee, WI, USA in use in our department from 2004 to 2018) either in two-dimensional (ST2D) or three-dimensional mode (ST3D), Discovery MI digital (GE Healthcare, introduced in our department in 2018) (MI), Discovery MI DR (GE Healthcare, 2018) (MIDR), Guardian Body Mobile Unit (Philips Medical Equipment, is use from 2016 to 2018) (MU). The data were reconstructed with OSEM, with a different number of iterations (2, 5 and 4 iterations, respectively and unknown for MU). For MI and MIDR only the options Time of Flight (TOF) and Point Spread Function (PSF) were available and were always used. Z-axis filtering was applied. Starting January 2019, intravenous iodine contrast media was injected unless contraindicated. All PET/CT systems underwent periodical quality control including calibration of the Standard Uptake Value in keeping with the vendor’s maintenance schedule.

### SUV and size measurements

All SUV and size measurements were done by two board-certified nuclear medicine and radiology specialists, using a Syngo.via™ post-processing workstation (Siemens Healthcare GmbH, Forchheim, Germany, VB10B 2015). To ensure the lack of inter-observer variability, a pilot study on 15 studies was done by consensus reading between the two readers. For another sample of 10 studies, measurements were done independently by the two physicians among which no difference greater than 10% was found in any of the SUV and size measurements. Further measurements were done independently by either of the two imaging specialists.

The SUVmax in the hottest cervical lymph nodes in level II or III (SUVmax_CLN_) was obtained for all the included studies. When the uptake in the cervical lymph nodes was not higher than cervical background activity, the SUV in the largest level II or III cervical lymph node seen on CT was reported as SUVmax_CLN_. SUVmax in the ipsilateral palatine tonsil (from now on designed as ‘SUVmax tonsil’) and mean liver uptake (from now on ‘SUV mean liver’) were also obtained. SUV mean liver was measured as the SUVmean in a spherical VOI in the right lobe of the liver of 30cm^3^ (+/- 3cm^3^) volume, a measure less noisy than SUVmax, in keeping with the liver uptake measurement previously used in the Euronet-2 protocol [9]. The ratio of SUVmax_CLN_ to SUV mean liver was compared to threshold values 1.3 and 2, corresponding roughly to Deauville score 4 and 5 respectively in early response assessment of Hodgkin lymphoma [9].

Lymph node size was measured on axial CT slices as the short axis in millimeters.

### Statistical analysis

Statistical analysis was performed using Stata MP version 16.1 (StataCorp, College Station, Texas 77,845 USA). Coefficients of linear regression models are estimated in ordinary least squares.

Univariate estimations were conducted, where the predictor was a single variable among the following: SUVmax tonsil, SUVmean liver, size of the hottest cervical lymph node (imaging variables); age and sex (demographic variables); treatment status, PET/CT systems (PET/CT machine individually ST2D, ST3D, MI MIDR or MU) and grouping data for machines using TOF and PSF (MI and MIDR on the one hand, and ST2D, ST3D and MU on the other.)

Multivariate estimations were conducted with two or more of the previous predictors. For each predictor, the report includes the estimated coefficient, 95% confidence interval and the p-value. P value < 0.05 is considered to indicate statistical significance.

## Results

### Values of SUVmax_CLN_

191 PET/CT studies were included in the study. Table 2 reports the summary statistics of all the variables we considered for that sample of observations. We included PET/CT studies regardless of whether they were conducted before (37 out of 191), during (80) or after (74) treatment. In 56% studies, the patient was female. The youngest patient was 3.7 months old and the oldest was 20.8 years old. Mean SUVmax_CLN_ value in the overall population was 2.2 ± 1.3. For 136/191 (71%) of the PET/CT studies, the uptake in some cervical lymph nodes was above the cervical background uptake; for them, mean SUVmax_CLN_ value was 2.6 ± 1.2. From now on we include all studies whether the uptake is above or equal to background.

**Table 2:** Population characteristics and PET/CT results

Patient characteristics	No. (%)
**Number of studies**	191
**Number of patients**	76
**Primary tumor**	
Hodgkin Lymphoma	15
Non-Hodgkin lymphoma	22
Rhabdomyosarcoma	22
Ewing sarcoma	54
Osteosarcoma	14
Yolk sac tumor	15
Other solid tumors	49
**Treatment status**	
Before treatment	37
Active treatment	80
Follow-up	74
**Technical parameter**s	
ST2D	47
ST3D	16
MI	97
MIDR	25
MU	6
TOF and PSF	122
No TOF and no PSF	69
**Demographic parameters**	
Age in years	11.3 ± 5.5
Min Age	0.3
Median Age	12.1
Max Age	20.8
Female	109 (56.8)
Age in years	11.3 ± 5.5
Female	109 (56.8)
**Imaging parameters**	
SUVmax_CLN_	2.2 ± 1.3
SUVmean Liver	1.5 ± 0.50
SUVmax_CLN_ / SUVmean Liver	1.64 ± 0.96
<1.3	95 (49.8)
1.3–2	43 (22.5)
>2	53 (27.8)
SUVmax tonsil	5.7 ± 2.8

The numbers show repartition per study (not per patient). Data are shown as mean ± standard deviation or n (%).

### Univariate analysis

**Fig. 1:**
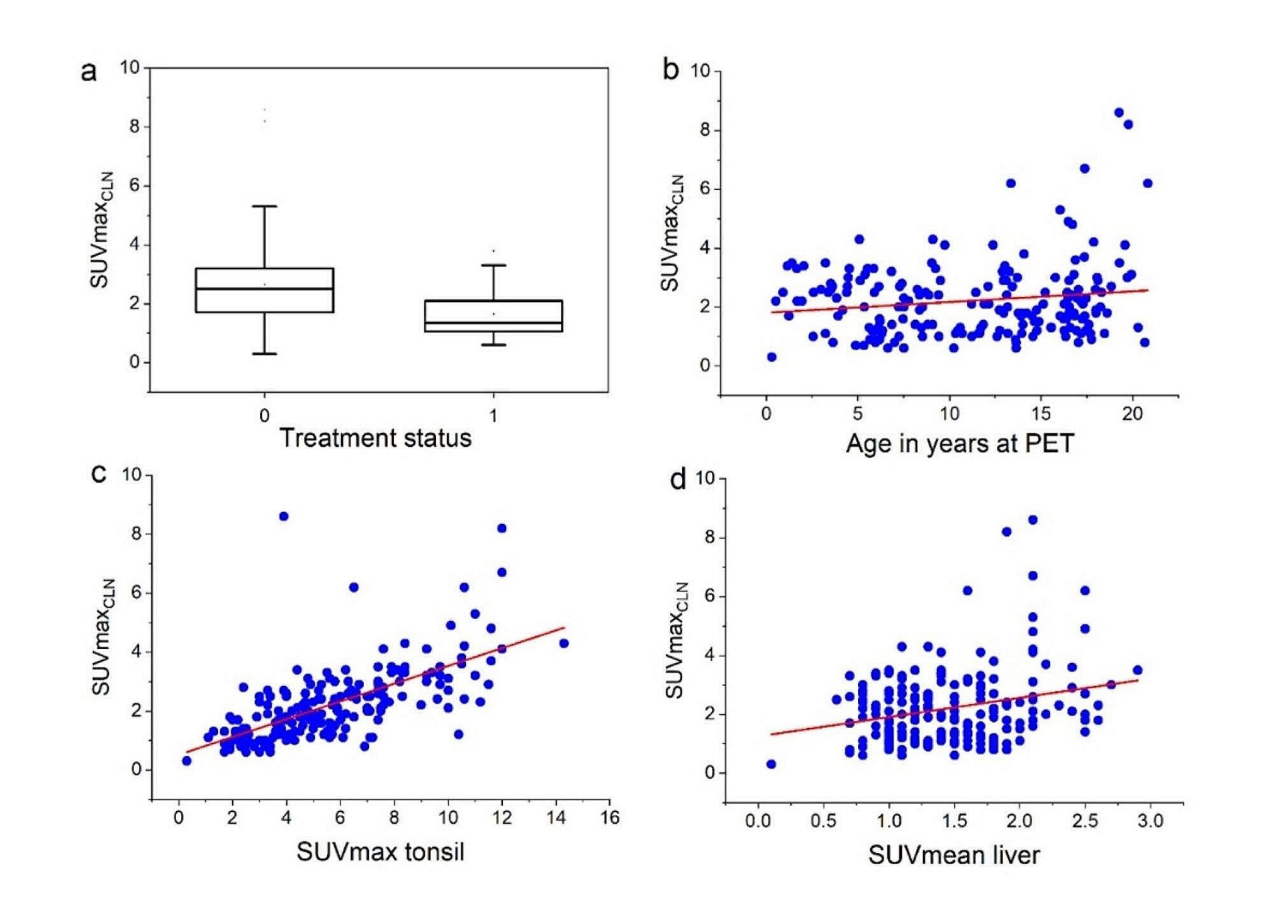
Predictors of the uptake in the hottest non-malignant cervical lymph node. a: treatment status (0: before treatment and follow-up, 1 = active treatment), b: age in years at time of PET/CT, c: SUVmax tonsil, d: SUVmean liver

The results of the univariate analysis are shown in Table 3; Fig. 1. All imaging variables studied (SUVmax tonsil, SUVmean liver, size of the hottest cervical lymph node) had a statistically significant relationship with SUVmax_CLN_. Treatment status was a significant predictor of cervical lymph node uptake (*p* < 0.001), showing lower values for patients under active treatment, and higher values during follow-up. No statistically significant effect of gender or type of machine was estimated, even grouped by technology (TOF and PSF together).

**Table 3:** Results of univariate linear regression analyses of the association between demographic, technical and imaging predictors on cervical lymph nodes uptake (SUVmax_CLN_)

Predictor	CE	*p*-value	*R* ^2^	[95%CI]	Intercept
**SUVmax tonsil**	0.30 *	< 0.001	0.432	[0.25,0.35]	0.52
**SUVmean liver**	0.66 *	< 0.001	0.069	[0.31,1.00]	1.25
**Lymph node size (mm)**	3.23 *	< 0.001	0.384	[2.64,3.82]	0.23
**Treatment status**: **Before treatment**	0.15	0.50	0.002	[-0.30;0.61]	2.18
**Treatment status**: **Active treatment**	-0.99 *	< 0.001	0.148	[-1.33, -0.65]	2.63
**Treatment status**: **Follow-up**	0.92 *	< 0.001	0.123	[0.57, 1.27]	1.86
**Age in years**	0.036 *	0.03	0.025	[0.003,0.069]	1.8
**Gender: Female**	-0.051	0.786	< 0.001	[-0.42,0.32]	2.24
**PET/CT machine: MI**	-0.070	0.707	0.001	[-0.43, 0.29]	2.25
**PET/CT machine: MIDR**	0 0.015	0.955	< 0.001	[-0.53; 0.56]	2.21
**PET/CT machine: ST2D**	0.36	0.09	0.015	[-0.059;0.78]	2.12
**PET/CT machine: ST3D**	-0.38	0.258	0.007	[-1.03; 0.28]	2.25
**PET/CT machine: MU**	-0.74	0.164	0.010	[-1.78; 0.30]	2.24

Each row shows the results of a different regression on a single variable. CE = coefficient estimate. CI = confidence interval. *p-value < 0.05

Age had a weak but statistically significant relationship with SUVmax_CLN_, showing higher values for older patients. Since the regressors may be correlated one with another, we cannot infer whether any of those is an independent predictor of SUVmax_CLN_. The comparison of R^2^ of the predictors however suggests that SUVmax tonsil is the best single linear predictor of SUVmax_CLN_. In particular, it is a better single predictor than SUVmean liver.

### Multivariate analysis

Would a combination of part or all of the regressors be a better predictor than SUVmax tonsil alone? To address this question, we conducted a multivariate linear analysis including both SUVmax tonsil and other relevant regressors. Of note, the size of the cervical lymph node was not considered as a potential explanatory variable in the multiple linear regression. Indeed, the size of a node is influenced by malignant involvement and as such does not meet the requirements for a predictive tool of benign uptake values.

The results are reported in Table 4. None of the predictors identified in the univariate analysis has an effect on cervical lymph node uptake statistically significant at 5%. SUVmax in the tonsil, however, was still significant, and the value of its coefficient had only little variation across different regressions. These results confirm that SUVmax in the tonsil is the best linear predictor, and imply that it can be used to predict the expected value of SUVmax_CLN_ without considering any other variable.

**Table 4:** Results of multivariate regression analyses of the association between SUVmax_CLN_ and imaging, demographic and technical variables

Predictor	Model A	Model B	Model C	Model D	Model E	Model F
**SUVmax tonsil**	**0.290** * [0.24,0.34] (*< 0.001)*	**0.282** * [0.22,0.34] *(< 0.001)*	**0.298** * [0.25,0.35] *(< 0.001)*	**0.305** * [0.26,0.36] *(< 0.001)*	**0.303** * [0.25,0.35] *(< 0.001)*	**0.260** * [0.20,0.32] *(< 0.001)*
**SUVmean liver**	0.209 [-0.07,0.49] *(0.191)*					0.388 [-0.07,0.85] (0.096)
**Treatment status: Active treatment**		-0.09 [-0.49,0.31] *(0.654)*				-0.145 [-0.56,0.27] (0.493)
**Treatment status: Follow-up**		0146 [-0.24; 0.53] (0.455)				0.218 [-0.19,0.62] (0.290)
**Age in years**			-0.009 [-0.02,0.04] (0.470)			-0.016 [-0.06,0.03] (0.448)
**Gender: Female**				-0.226 [-0.50,0.052] *(0.110)*		-0.281 [-0.58,0.01] (0.062)
**PET/CT** **machine: MI**					0.449 [-0.36,1.25] (0.272)	0.576 [-0.23,1.39] (0.163)
**PET/CT** **machine: MIDR**					0.537 [-0.33,1.41] (0.225)	0.606 [-0.27,1.48] (0.174)
**PET/CT** **machine: ST2D**					0.335 [-0.50,1.17] (0.429)	0.546 [-0.30,1.39] (0.203)
**PET/CT** **machine: ST3D**					0.276 [-0.64,1,19] (0.552)	0.504 [-0.44,1.44] (0.292)
**R** ^**2**^	0.438	0.437	0.433	0.439	0.438	0.464

Each column shows the results of a different regression, the regression of SUVmax_CLN_ on SUVmax tonsil and one or more other predictors (A: SUVmean liver; B: Treatment status [reference group: Treatment status: Before treatment]; C: Age; D: Female [reference group: Male]; E: Machine [reference group: PET/CT machine = MU]; F: all previous predictors). Results are shown as coefficient estimate, confidence interval and p-value. *p < 0.05

From Table 3, and the estimations of the standard errors of the estimates, the 95% CI of SUVmax_CLN_ can be predicted from the following formula:

$$\begin{gathered} Expected\,{\text{SUVmax}} = 0.52 + 0.3\,{\text{*}}\,SUVmaxtonsil \\ \pm 1.96\sqrt {{{\left( {0.025\,*\,SUVmaxtonsil} \right)}^2} + 0.02} \\ \end{gathered}$$

We conducted two additional estimations to address potential limitations of the previous results. First, the multivariate estimations of Table 4 do not include control variables for the type or location of tumor. To rule out that including these variables would change our results, we estimated a fixed-effects model of the linear regression of SUVmax_CLN_ on SUVmax tonsil, with individual fixed effects. Individual fixed effects control for the type and location of tumor, as well as for any other constant individual characteristic. The estimated coefficient for this model was 0.329 (95% CI: [0.246,0.412], p-value < 0.001, R2 = 0.711) if no additional predictor other than SUVmax tonsil was included, and 0.274 (95% CI: [0.167,0.382], p-value < 0.001, R2 = 0.711) if all regressors of Model F were included. Second, to check whether our results may apply to adults, we ran estimations from Model A to F for the subsample of 62 patients aged 16 or more in our sample. The estimated coefficient for this model was 0.376 (95% CI: [0.256,0.496], p-value < 0.001, R^2^ = 0.395) if no additional regressor other than SUV max tonsil were included, and 0.400 (95% CI: [0.236,0.564], p-value < 0.001, R2 = 0.505) if all regressors of Model F were included.

These estimations are close to the estimations of SUVmax tonsil in Table 3 and in Models A to F of Table 4, which show that the statistically significant predictive effect of tonsillar uptake on SUVmax_CLN_ is not substantially affected by intrinsic individual characteristics, and remains true for teenagers and young adults.

### Ratio SUVmax_CLN_ /SUVmean liver

**Fig. 2:**
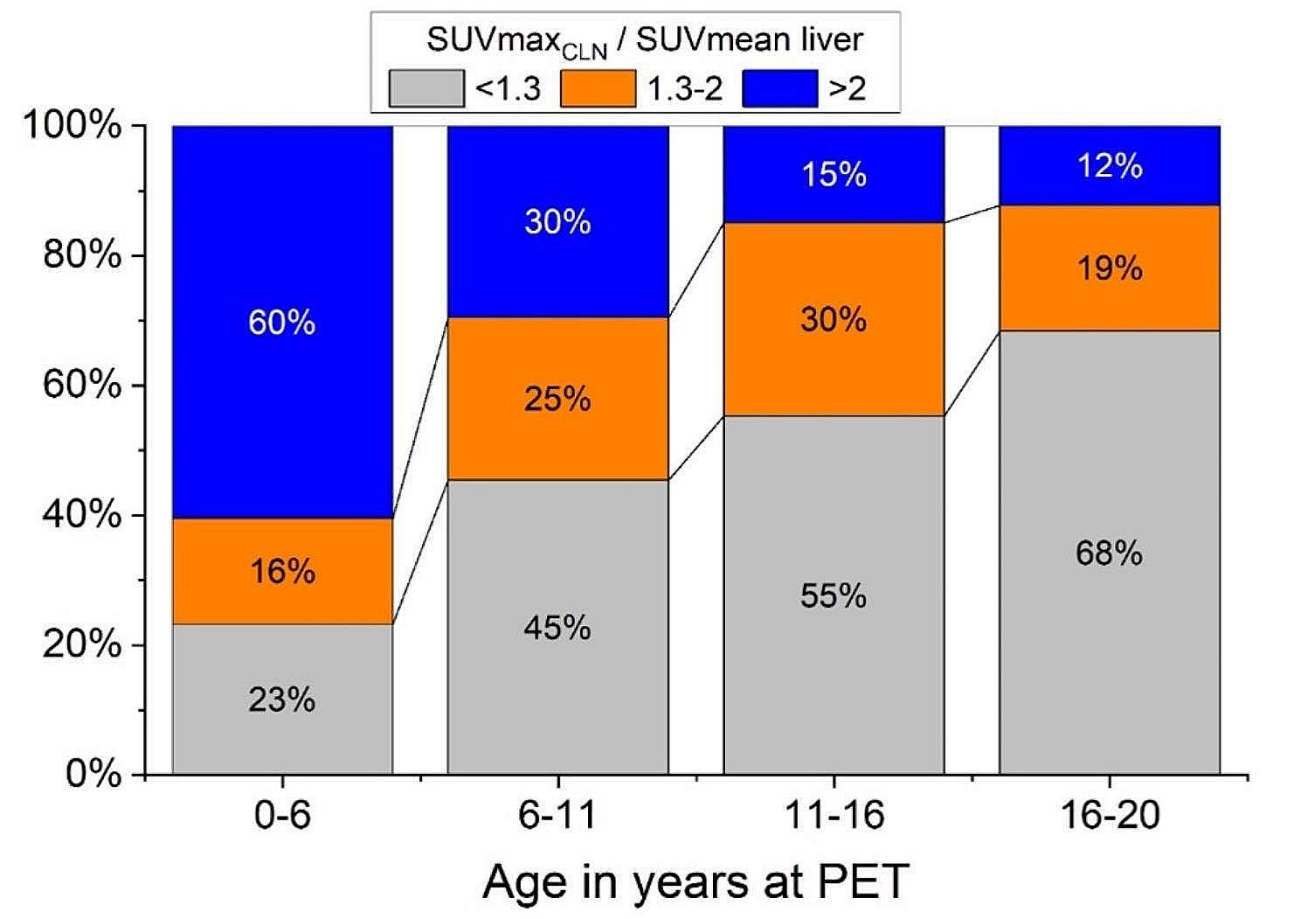
Repartition of the ratio SUVmax_CLN_ /SUVmean liver per age groups

The ratio SUVmax_CLN_ /SUVmean Liver was above 1.3 for 95 (50%) studies and above 2 for 53 (28%) studies (Table 2). Table 5 shows the repartition of SUVmax_CLN_, mean liver uptake and their ratio by age groups. Younger patients had significantly lower liver SUVmean. In consequence, the ratio SUVmax_CLN_ /SUVmean Liver tends to be higher for younger children, especially below 10-year-old and even lower below 6-year-old (Fig. 2). In the age group 0–6, 33/43 (77%) of the studies had a ratio above 1.3. For ages 6–10 years, the proportion was 23/44 (52%).

**Table 5:** SUVmax_CLN_, SUVmean in the liver and their ratio by age group

	Age group				
	A: 0–6 y.o.	B: 6–11 y.o.	C: 11–16 y.o.	D: 16–20 y.o.^$^	
**Number of studies**	43	44	47	57	
**SUVmax**_**CLN**_	2.23 ± 0.98	1.86 ± 0.93	2.02 ± 1.05	2.64 ± 1.71	
**SUVmean liver**	0.984 ± 0.30	1.19 ± 0.25	1.59 ± 0.35	1.97 ± 0.40	
**SUVmax**_**CLN**_ **/ SUVmean liver**	2.40 ± 1.08	1.62 ± 0.86	1.32 ± 0.69	1.34 ± 0.80	
Δ*		**B-A**	**C-B**	**D-C**	
**Δ SUVmax**_**CLN**_ **p-value**		− 0.371 0.177	0.164 0.545	0.612 0.104	
**Δ SUVmean liver** **p-value**		0.203 ** 0.010	0.401 ** < 0.001	0.374 ** 0.002	
**Δ SUVmax**_**CLN**_ **/ SUVmean liver** **p-value**		− 0.781 ** 0.009	− 0.298 0.186	0.0187 0.919	

Results are shown as mean ± standard deviation. * Δ indicates the difference of the means between two successive age groups. ** *p* < 0.05. ^$^ Group D includes patients aged 16 to 20 (included)

### Intrasubject variability

**Fig. 3:**
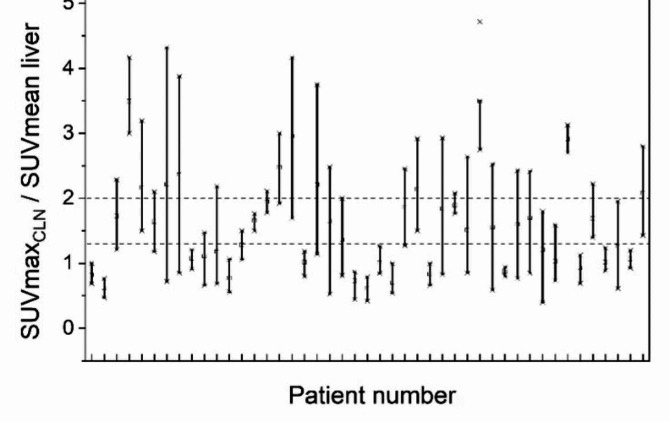
Spread of values of SUVmax_CLN_ /SUVmean liver for patients who did more than one PET/CT. The two horizontal dotted lines indicate the benchmark values 1.3 and 2

44 patients had two or more PET/CT studies. There was a high variability of the ratio SUVmax_CLN_ /SUVmean Liver (Fig. 3). Specifically, more than 40% of patients had values that flipped above and below 1.3 across serial PET/CT studies.

## Discussion

To the best of our knowledge, this study is the first to show a strong correlation between the SUVmax in the hottest non-malignant cervical lymph node and the SUVmax in the ipsilateral palatine tonsil. This result is not surprising. Increased uptake in the palatine tonsils may be the result of an infectious process involving the Waldeyer ring, in which satellite adenopathy is common. Our study was done in the pediatric population, since we assumed that the prevalence of benign increased uptake neck lymph nodes would be higher. However, the data for the 16–20 years old show the same correlation with slightly higher values of uptake in the cervical nodes, suggesting that this result may remain true for adults. Of note, we have found no study showing a correlation between the size of non-malignant cervical lymph nodes and the size of the ipsilateral palatine tonsil.

**Fig. 4:**
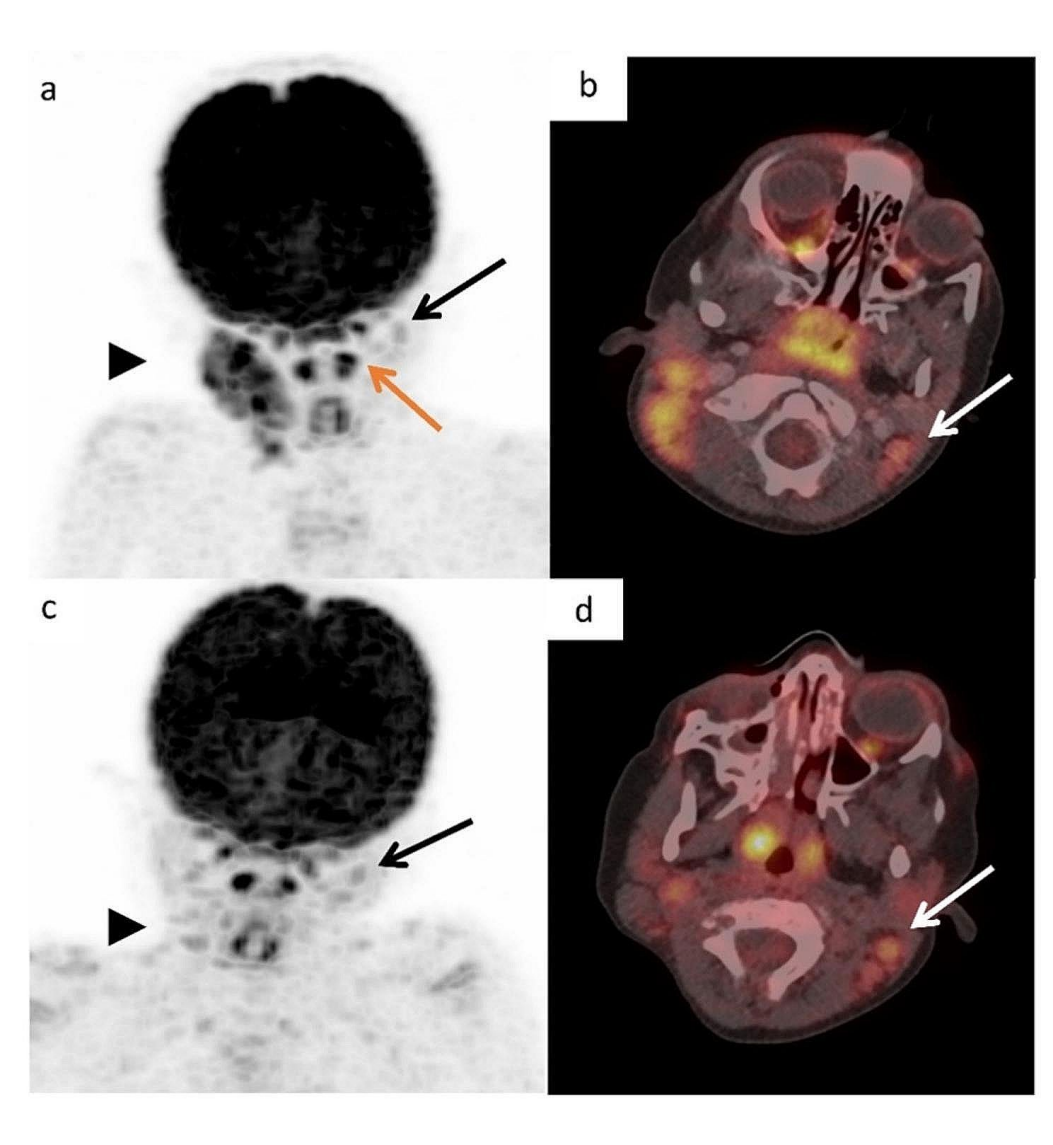
An example of the incremental value of the results of the study. A three-year old boy presented with right neck lymphadenopathy. Biopsy showed classical Hodgkin lymphoma. PET/CT at staging (a, b) showed right neck hypermetabolic lymph nodes SUVmax 12.3 and slightly enlarged nodes in the left neck (black arrow) with moderately increased uptake SUVmax 3.5. There was strong uptake in the left palatine tonsil (SUVmax 10.1, orange arrow), but no clinical or imaging evidence for tonsillar lymphomatous involvement. After two cycles of chemotherapy (c, d), the uptake in the right cervical lymph nodes decreased to SUVmax 3.5 (arrowhead) and the uptake in the left cervical nodes remained stable at SUVmax 3.5. SUVmean liver was 1.4. Assuming that the left palatine tonsil is not involved, the formula presented in the Results section predicts a SUVmax value of 3.5$\pm$ 0.6 for non-malignant nodes, which was exactly the measured uptake. At early response assessment, the ratio SUVmax/SUVmean liver was 2.4. Per Deauville criteria, this is an inadequate response to treatment, although this is the 20th percentile of the normal values in this age group, and equal to the uptake value in the left cervical nodes

The formula given for the prediction of SUVmax in benign cervical lymph nodes may be useful for the staging of pediatric malignancies that may involve cervical lymph nodes, such as rhabdomyosarcoma and Hodgkin lymphoma when tonsils are not involved (see an example in Fig. 4).

For pediatric Hodgkin lymphoma, neither the EuroNet-PHL protocol [10] nor the NCCN guidelines (Version 1.2022) [11] provide objective criteria for positivity of lymph nodes at staging. (The NCCN guidelines misleadingly show the Deauville criteria in the “Principles of staging” section page 31, although these criteria are validated for early response assessment only after two courses of chemotherapy). Considering cervical lymph nodes at staging as PET-positive is consequently a subjective decision of the interpreting physician, with potential inter-observer variability. Using the results presented here, the interpreting physician can compare the observed SUVmax to the predicted expected value of SUVmax of a benign/reactive node, computed from the uptake value in the non-involved tonsil, in order to decide whether the lymph node is positive or not.

The results presented here may also be apply to solid tumors. For instance, in childhood rhabdomyosarcomas, the rare lymph node involvement at diagnosis classifies the patient as high-risk, with consequences for the treatment plan [12]. By comparing cervical lymph node uptake to tonsillar uptake, the need for lymph node biopsy may be waived in certain cases.

At the end of treatment too the results presented here may alleviate the need for biopsy and justify a more watchful management in the case of low-grade residual uptake in cervical lymph nodes, when the uptake value in the tonsil makes it consistent with a benign etiology.

Moreover, the fact that after adjusting for tonsillar uptake, no correlation is seen between liver uptake and non-malignant cervical lymph nodes uptake suggests that tonsillar uptake may replace liver uptake as a reference value for response assessment of Hodgkin lymphoma involving cervical lymph nodes.

The use of tonsillar uptake as a reference depends upon the low incidence of tonsillar involvement by pediatric malignancies. For Hodgkin lymphoma, Waldeyer ring involvement is extremely rare in the pediatric population with only a few case reports published [13]. For non-Hodgkin lymphomas, Burkitt lymphoma and diffuse large B-cell lymphoma may involve the Waldeyer ring generally with clear clinical symptoms [14]. In these rare cases tonsillar uptake cannot be used as reference. Symmetry of the uptake in both palatine tonsils has been proposed as a predictor of non-malignant uptake in the adult population [15].

Previous published works have reported the occurrence of increased uptake in non-malignant cervical lymph nodes, including in the pediatric population [16]. included 27 pediatric patients after treatment for lymphoma with increased uptake in cervical lymph nodes, all of them with disease-free survival during follow-up, and 3 of them with histologic evidence of benign follicular hyperplasia. An adult study [17] included 87 patients after treatment for diffuse large B-cell lymphoma with increased uptake in cervical lymph nodes, 9% of whom ultimately had malignancy. In the latter study, the authors found no correlation between the unilateral pattern of cervical lymph.

node uptake and the risk of malignancy. The mean SUVmax in these nodes was not reported in these two papers, so a direct comparison with our population is not feasible. In [18], the authors studied in a systematic fashion the frequency of increased uptake in cervical lymph nodes in children without head and neck cancer. For the subgroup of 38 patients that were proved to be free of disease, the authors reported a mean SUVmax of 2.1, as compared to 2.9 in our study when limiting the analysis to the 69 studies performed on PET/CT scanners with similar technology (non-PSF and non-TOF), but their PET/CT system (a Gemini Philips hybrid scanner installed in 2007) was different from those used in our study and their population size was smaller.

The challenges posed by “hot” cervical lymph nodes of unknown significance have been addressed in other ways. Texture analysis [19] and artificial intelligence [20] have been shown to improve the diagnostic accuracy of FDG PET/CT in the lymphatic staging of oral squamous cell carcinoma in adult patients. Other tracers such as Gallium 68 (^68^Ga)–labeled fibroblast-activation protein inhibitor (FAPI) have been sporadically reported to distinguish reactive lymph nodes from tumor metastatic lymph nodes in a patient with nasopharyngeal carcinoma [21]. Further studies that would include the results presented here as input to train artificial neural networks for the adequate classification of cervical lymph nodes on PET/CT studies would be of interest.

On a technical level, we did not observe any influence of the use of TOF and PSF technologies on the values of uptake of benign cervical lymph nodes. This result differs from the conclusion of several studies that have highlighted the influence of TPF and PSF technologies on the values of SUV for small lesions [22, 23]. A possible explanation for this finding is the relatively low values of uptake in the cervical lymph nodes in our study. For example, in reference [22] the authors studied the influence of TOF and PSF on the value of uptake of lung tumors and observed an increase of SUVmax value of 26%. However, the lesions they studied had an average SUVmax of 7.2 without TOF and PSF, as compared to 2.9 in our study.

Present study also showed that in the pediatric population the uptake value of the hottest non-malignant cervical lymph node is frequently above the liver SUVmean. To the best of our knowledge this observation as well has not been previously reported. This ratio is used for early response assessment of pediatric Hodgkin lymphoma both and in the Euronet-2 protocol [10], and in the American guidelines published by the National Comprehensive Cancer Network (NCCN) [11]. In these protocols, PET/CT is performed after two cycles of chemotherapy. Patients with low risk classical Hodgkin disease are considered to have an inadequate response (IR) to treatment if the uptake in residual lymphoma is higher than 1.3 times the average liver uptake, corresponding to a Deauville score 4 in previous works that were based on visual assessment. These patients only are referred to radiotherapy. No particular attention is given to the age of the patients or to the body area involved by the lymphoma. Since Deauville score of 4 is a common finding in benign cervical lymph nodes, the proportion of patients with IR is expected to be higher in Hodgkin lymphoma involving the neck area than in other body areas, although it is not known to be a pejorative predictive factor [24]. Moreover, physiologic liver uptake increases with age, as observed in our cohort. This result has already been reported [25] although its implications on the Deauville score has not been studied. It implies that younger patients are at higher risk of being classified as IR, despite having a better prognosis [26].

### Limitations of the study

This study has several limitations. It is a single center study, and although we pooled the studies of four different PET/CT machines and did not find differences between them, the applicability of these results to other departments is not known.

An intrinsic limitation stems from the methodology of this study. Formally, our methodology of describing the distribution of non-malignant cervical lymph nodes only allows an interpreting physician confronted with a “hot” cervical lymph node to know whether a benign condition may explain such an uptake value. This is a different question than whether such an uptake value is suspicious for malignancy. However, it allows to have a large study population. For the latter purpose, a large cohort of studies with involved cervical nodes is necessary, which is not readily available in clinical practice. Moreover, biopsy is done in general in a single site of disease, chosen for being accessible and unambiguous. As a consequence, uptake values in the sites of disease chosen for biopsy are higher than other potential sites of disease. This may lead to overestimate the uptake values of true sites of disease.

Another limitation is the applicability of this work to cervical lymph nodes stations that were involved with Hodgkin lymphoma at presentation. Present study has assessed the uptake in non-malignant cervical lymph nodes, showing relatively high level of uptake in these areas in a large number of patients and correlation to tonsillar uptake. Whether these results are true for lymph nodes that were previously involved with Hodgkin disease is not known and should be assessed in a suitable patient cohort.

For comparing the uptake in cervical lymph nodes to the liver uptake, we used the definition of liver uptake as Hasenclever et al. [9] but a different definition of the uptake in the target lesions. The authors used SUV peak in the hottest voxel and the three hottest adjacent ones, while we used SUVmax, which by definition is higher than SUVpeak. However, their definition of SUVpeak depends on the size of the voxel, which depends on the PET/CT scanner. Moreover, their definition of SUVpeak requires a dedicated software and is not measurable with standard workstations. It is not clear why the authors did not use SUVmax as a measure of residual uptake, as is common in other studies [27].

## Conclusion

Increased FDG uptake in cervical lymph nodes is a common physiologic variant in the pediatric population. Ipsilateral palatine tonsil SUVmax is the best predictor of the SUVmax of benign cervical lymph nodes. This result may help to avoid over-staging of malignancies that may spread to the cervical lymph nodes. In doubtful cases, biopsy should be prompted.

For early response evaluation to treatment in lymphoma involving the cervical lymph nodes, further studies that would take the tonsillar uptake into account are warranted.

## Data Availability

The datasets used during the current study are available from the corresponding author on reasonable request.
